# Supplementary material for: Regulating the glucose-6-phosphate dehydrogenase encoding gene gsdA and its impact on growth and citric acid production in Aspergillus niger
Source: PLoS One. 2025 Apr 24;20(4):e0321363. doi: 10.1371/journal.pone.0321363 (PMC12021212; doi:10.1371/journal.pone.0321363)
Supplement: S1 File — (PDF) [file pone.0321363.s001.pdf]

S1 File. Coding sequence of *gsdA_mutPAM* in pSF8 and pSF503.

>gsdA_mutPAM
ATGGCCAGCACAATAGCACGCACTGAGGAACGCCAGAATGCTGGGTGAGTTTTGCGGTCTGCCTCTCTGACATCACACCC
CTCCTCCCACTCCCGTCCCTCCTGCCCGCCGCCCAGACGTGAGGATTCACCACCCACGACTCTCCATAACAAGCCCCGTC
GCCCAAACATTCACTGGCAGGCTTCCCGCTTTCCATTATTCTTCAATTCGTCACCAGGATTACTCCTTCGGGCTTAACGAAA
GGACTATCCTTCTGACTCACCACCAACCCTCACTGCCCCTCCTGCATGCTGTAGCGGACTGCGGGCACCGACCTGCATCA
TCGATCTACACCCGATCCCTGTGAACCGTCTGTAAATATTCGTCAAGCTATAGCCTAGCTAACATGGATGTTTTACGTAGCA
CCATGGAGCTCAAAGATGACACTGTCATCATAGTACTGGGTGCCTCCGGAGATCTTGCAAAGAAGAAAACCGTCAGTGACG
ACCCCCTGATTCATGTTGACCTGACAGAAAGCTAACCTTTTACAGTTCCCGGCCCTTTTCGGCCTTGTATGTCCTCTCCCAG
ATCCAATTGCAGTTTGACTCACCAGTATGGTTGCTGATTTGCGCTTCCAGTATCGCAACAAGTTCCTCCCCAAGGGAATCAA
GATCGTCGGATATGCCCGGACAAACATGGACCATGAGGAGTACCTGAGGCGTGTGCGCTCATACATCAAGACCCCTACCA
AGGAAATCGAAGAGCAGCTGGACAGCTTCTGCCAGTTCTGCACCTACATTTCCGGTCAATATGACAAGGATGACTCGTTCA
TCAACCTCAACAAGCACCTCGAGGAGATTGAGAAGGGCCAGAAGGAGCAGAACAGAATCTACTACATGGCCCTCCCtCCCA
GCGTTTTCACCACCGTTTCCGACCAACTTAAGCGCAACTGCTACCCCAAGAACGGCGTTGCCCGTATCATCGTGAGTCAAT
CCTGGGCTGGTATCACCCTGCCATTGGTCATTATTCTTACTCGCTTGTTTTCCTATTTCACAGGTAGAGAAGCCTTTCGGCA
AGGACCTTCAGAGCTCGCGCGATCTCCAAAAAGCCCTGGAGCCTAACTGGAAGGAAGAGGAGATCTTCCGTATCGACCAC
TACCTGGGTAAGGAGATGGTCAAGAACATCCTTATCATGCGCTTCGGAAACGAATTCTTCAACGCCACCTGGAACCGTCAC
CACATCGATAACGTTCAGGTACGACCTTGCGCTATCCAATTGGCCTATTGATTTACTTGCTAAATTGTCGCTTCTATCATTAG
ATCACATTCAAGGAGCCCTTCGGCACTGAGGGACGTGGTGGTTACTTCGATGAATTCGGCATCATCCGTGATGTCATGCAG
AACCGTACGTTCAAAGTCACGCTCGACATCTCCGACATGATGCTGATAAAAATCTCTCCTAGACCTTCTCCAGGTGTTGACG
CTGCTCGCTATGGAGCGCCCCATTTCCTTCTCCGCCGAGGACATCCGTGACGAGAAGGTACAGTGTGCGCTTGACTATTG
GTTGTGCTGGGTTACTGACACTTAACCAGGTTCGTGTCCTCCGTGCGATGGACGCCATTGAGCCCAAGAACGTCATTATTG
GCCAGTACGGAAAGTCTCTGGATGGCAGCAAGCCCGCCTACAAGGAGGACGAAACCGTTCCCCAGGATTCCCGCTGCCC
CACCTTCTGCGCTATGGTCGCCTACATCAAGAACGAGAGGTGGGACGGTGTTCCTTTCATCATGAAGGCTGGCAAGGGTAT
GTACCTCTTTCCAAGCGATCATAGCACCGATTGGTATACTAATAATTCGCAGCCTTGAACGAGCAGAAaACCGAGATCCGTA
TCCAGTTCCGTGACGTTACCTCCGGAATTTTCAAGGACATCCCTCGCAACGAGCTCGTTATCCGCGTCCAGCCCAACGAGT
CCGTGTACATCAAGATGAACTCCAAGCTGCCTGGCCTGTCCATGCAGACGGTTGTGACTGAGCTCGACCTCACCTACCGC
CGCCGCTTCTCCGACCTCAAGATCCCCGAAGCCTACGAGTCTCTGATCCTGGATGCTCTGAAGGGCGACCACTCCAACTT
CGTCCGTGACGATGAGCTGGATGCCAGCTGGAGGATCTTCACCCCTCTCCTGCACTACCTGGATGACAACAAGGAGATCA
TCCCCATGGAATACCCCTACGGTACGTGCACTTCTTGCAATTTGTCTAAATCGCTTACATACTGACCAACGCGCAGGCTCCC
GCGGACCCGCCGTCCTTGATGACTTCACCGCGTCCTTCGGCTACAAGTTCAGCGATGCTGCTGGCTACCAGTGGCCCTTG
ACTTCCACCCCCAACCGTCTGTAA
