# Supplementary material for: Regulating the glucose-6-phosphate dehydrogenase encoding gene gsdA and its impact on growth and citric acid production in Aspergillus niger
Source: PLoS One. 2025 Apr 24;20(4):e0321363. doi: 10.1371/journal.pone.0321363 (PMC12021212; doi:10.1371/journal.pone.0321363)
Supplement: S2 File — (PDF) [file pone.0321363.s002.pdf]

S2 File. Result of Sanger sequencing the *pyrG* locus of SF387 and SF388 using primer P23.

>pyrG_SF387
CCGTGACTCGACTACTCTMTTATACAATCCTCTTTCCATTCCCGCATTAACCCCTCCATCAACACCATGTCCTCCAAGTCGC
AATTGACCTACACTGCCCGTGCCAGCAAGCATCCCAATGCTCTGGCCAAGAARACCAATGTGACTGTCTCRGCTGACGTTA
CCACCACTAAGGAGCA

>pyrG_SF388
GCRGTSACTTCGACTACTCTCATTATACAATCCTCTTTCCATTCCCGCATTAACCCCTCCATCAACACCATGTCCTCCAAGTC
GCAATTGACCTACACTGCCCGTGCCAGCAAGCATCCCAATGCTCTGGCCAAGAARACCAATGTGACTGTCTCGGCTGACGT
TACCACCACTAAGGAGCTACCTAAGGTTGCACCTCTTGYGSAGACGARYCTTCAAAGTAGACGGATATACACGCTAAAACA
AACTTGCT
