# Supplementary material for: Regulating the glucose-6-phosphate dehydrogenase encoding gene gsdA and its impact on growth and citric acid production in Aspergillus niger
Source: PLoS One. 2025 Apr 24;20(4):e0321363. doi: 10.1371/journal.pone.0321363 (PMC12021212; doi:10.1371/journal.pone.0321363)
Supplement: S3 File — (PDF) [file pone.0321363.s003.pdf]

# S3 File. Result of Sanger sequencing the native *gsdA* locus of strain SF395 using primers P25 to P28.

>gsdA_SF395
CTGTGACATTATATTCGTCAAGCTATAGCCTAGCTAACATGGATGTTTTACGTAGCACCATGGAGCTCAAAGATGACACTGT
CATCATAGTACTGGGTGCCTCCGGAGATCTTGCAAAGAAGAAGACCGTCAGTGACGACCCCCTGATTCATGTTGACCTGAC
AGAAAGCTAACCTTTTACAGTTCCCGGCCCTTTTCGGCCTTGTATGTCCTCTCCCAGATCCAATTGCAGTTTGACTCACCAG
TATGGTTGCTGATTTGCGCTTCCAGTATCGCAACAAGTTCCTCCCCAAGGGAATCAAGATCGTCGGATATGCCCGGACAAA
CATGGACCATGAGGAGTACCTGAGGCGTGTGCGCTCATACATCAAGACCCCTACCAAGGAAATCGAAGAGCAGCTGGACA
GCTTCTGCCAGTTCTGCACCTACATTTCCGGTCAATATGACAAGGATGACTCGTTCATCAACCTCAACAAGCACCTCGAGGA
GATTGAGAAGGGCCAGAAGGAGCAGAACAGAATCTACTACATATGATAACTTCGTATAGCATACATTATACGAAGTTATCCC
ATGCTAATCATATAACGATCTTTATTATAGATATGCCATCGCCGTTTCGCTAGGCGCAATCCCTGTCCTGGGATTCATCCAT
GGTGTCCTCGTCGGCTCTTTTCGCAAGGCCGCTGGCGTGCCGTACCCCCACGCCTATGCCAGCATTGAGCAATGTAAAGC
TAACGTGCGTGAGCCCAAGAAACTAAATACCTATAGCAAAACAGATTGTGTTCCAAGAGAGAGTACTAAATGACGTTTGTGA
ACAGCCCAAAGCCTACAAATTCAACTGCGCACAACGCGCCCACGGCAACTTCCTCGAGAACGCGCCGCAGACAATGCTCT
CTATCCTGGTGGCAGGCGTCAAGTACCCAGAGGCAGCAGCGGGCTTAGGAGCGGCCTGGGTTGTTCTCCGCACCCTCTA
CATGCTGGGCTATATTTATAGCGACAAGCCGAACGGCACCGGCAGGTACAATGGTTCGCTGTACTTGCTTGCGCAAGCGG
GTCTTTGGGGATTGAGCGCATTTGGTGTTGCAAAGGATTTGATGTAAATGTAGTCGACATCTTAGCACAGAGGGGAGAGTT
GATAAAATGTGGTCTGTTTGAATGATAGTCGGGTTCGTGACCTATATTCGTGATAGTGGAGATAGGTCTGCGCCTATCTTAT
CGGGCCGGAGCAAAAATTCCACCGCAGCGGGGTGAGTTTTCGTTATACAGCCATCCCACTTCCAGCTTCAAATTGTCAGTT
TAATCCAGCCCAATTCAATCATTGGAGAACCGCCATCATGTCgTCGAAGTCCCACCTCCCCTACGCAATTCGCGCAACCAAC
CATCCCAACCCTTTAACATCTAAACTCTTCTCCATCGCCGAGGAGAAGAAAACCAACGTCACCGTCTCCGCAGACGTTACTA
CTTCCGCCGAGCTCCTCGATCTTGCTGACCGTACATCCTGCACCAATGCCCCTCCAGGATAACAAATAGCTGATGCGTAGT
GAGTACAGGCCTAGGCCCCTATATCGCAGTTCTGAAAACCCACATCGACATCCTCACCGATCTCACCCCGTCGACCCTTTC
CTCGCTCCAATCCCTCGCGACAAAGCACAACTTCCTCATCTTTGAGGACCGCAAGTTCATCGACATCGGCAACACCGTGCA
AAAGCAGTACCACGGTGGCGCTCTCCGCATCTCCGAATGGGCACACATCATCAACTGCGCCATCCTGCCGGGCGAAGGGA
TCGTCGAGGCCCTCGCACAGACAACCAAGTCTCCTGACTTTAAAGACGCGAATCAACGAGGcCTCCTGATTCTTGCCGAGA
TGACGAGTAAGGGATCTCTTGCGACAGGGGAGTACACGGCACGCTCGGTTGAGTACGCGCGGAAGTATAAGGGGTTTGTG
ATGGGATTCGTGAGTACAAGGGCGTTGAGTGAGGTGCTGCCCGAACAGAAAGAGGAGAGCGAGGATTTTGTCGTCTTTAC
GACTGGGGTGAATCTGTCGGATAAGGGGGATAAGCTGGGGCAGCAGTATCAGACACCTGGGTCGGCGGTTGGGCGAGGT
GCGGACTTTATCATTGCGGGTAGGGGCATCTATAAGGCGGACGATCCAGTCGAGGCGGTTCAGAGGTACCGGGAGGAAG
GCTGGAAAGCTTACGAGAAAAGAGTTGGACTTTGAGTGTGAGTGGAAATGTGTAACGGTATTGACTAAAAGGGATCCATAT
GTTTATTGCAGCCAGCATAGTATTACCAGAAAGAGCCTCACTGACGGCTCTAGTAGTATTCGAACAGATATTATTGTGACCA
GCTCTGAACGATATGCTCCCTAATCTGGTAGACAAGCACTGATCTACCCCTTGGAACGCAGCATCTAGGCTCTGGCTGTGC
TCTAACCCTAACTAGACGATTGATCGCAGACCATCCAATACTGAAAAGTCTCTATCAGAGGAAATCCCCAACATTGTAGTAG
TCAGGTTCCTTTGTGGCTGGGAGAGAATTGGTTCGCTCCACTGATTCCAGTTGAGAAAGTGGGCTAGAAAAAAGTCTTGAA
GATTGGAGTTGGGCTGTGGTTAAGCCGGCTTTTATTGACCTTATCATTTAGCAAAATATGGGCAGTTGCTATCAGGACCACA
TACTCTACCCGAAGCTTAAAGGCAAAAAGAAATTCTGTATGTCCTGCGAATCAACATTCCTCGTGTTATATGAGCCCAAGGC
GCTGAACCAGGAATATTAGCTACGCTTGTGGCTCGCGAAGCAATGATACTCCCTTCTGAAGTGTGTATTGAGCTAGTTACAT
TAGTGGCACATCTTAACACCAGCACATTGGCATATTTAGGATACTATTGATAATGGAATTCAACTATCTTGCTTTATAGCCGA
CTACAGCTTCGGAACGCAATCCTTCTTTACGTAAATGTGAAAATGCTCTTAGACAGCTTGAAAGGCCAAAAAATCTCCCAGA
AAAAAAAAAGAGAATTAGAGAAAATCCAGTGGGTATATAGCTATGGATGCCCTCAATTATCCTGTATCTTCAGATGTTCCAC
GAGATCCACTTAGAACATAAGGCAATTCCTATCCTCACCATCTCATCTGTTTTGCTTCTCTTTAGGAAACACATGTTTCTACT
GACCTCGCCCCTTTCCTTGATCATTTCCACTGTCCAGTGATTGTCTCTAGAATTAGAGCTCTGATAACTTCGTATAGCATACA
TTATACGAAGTTATgAGGAGATCATCCCCATGGAATACCCCTACGGTACGTGCACTTCTTGCAATTTGTCTAAATCGCTTACA
TACTGACCAACGCGCAGGCTCCCGCGGACCCGCCGTCCTTGATGACTTCACCGCGTCCTTCGGCTACAAGTTCAGCGATG
CTGCTGGCTACCAGTGGCCCTTGACTTCCACCCCCAACCGTCTGTAAATAAGGGCGGTCGGCAGGTTATGACGGATGAGG
ATGAAAAAAAAAATTATTGCCAAAAAAGGCTAAAAAAAAGATGTTAATGCGATTGATTTTCGGTCGAGAATCATGGTATGACGG
GGCATCTGGGATGATATGACAGAAATGAAGCACTCGGGACTATTTATCGGTCGGCTGGGCAATAACTGGAGTTATCTATTC
GCAACCCCTTTTTAGAAACGAATGCAGAGAACGTAACGAACCACCCCCGGTCGGTTGGGACCGTCTCGTAAGGTAGAATTA
GGTTGGGAAAAGACAATCTAACCAATATACTTCCCCAGTTTCAGATTTGTTTTAATCCCCCGACCTTGCTTTAGGTCGTTCAC
ACCACCCGCTATTAATAAACAAGTACCGTCGCGGTGGTGGGTCTCAGCGTGGCCGGTGCCGATAAGACGAGGTACCTGA
