# Supplementary figures and images for: Regulating the glucose-6-phosphate dehydrogenase encoding gene gsdA and its impact on growth and citric acid production in Aspergillus niger
Source: PLoS One. 2025 Apr 24;20(4):e0321363. doi: 10.1371/journal.pone.0321363 (PMC12021212; doi:10.1371/journal.pone.0321363)

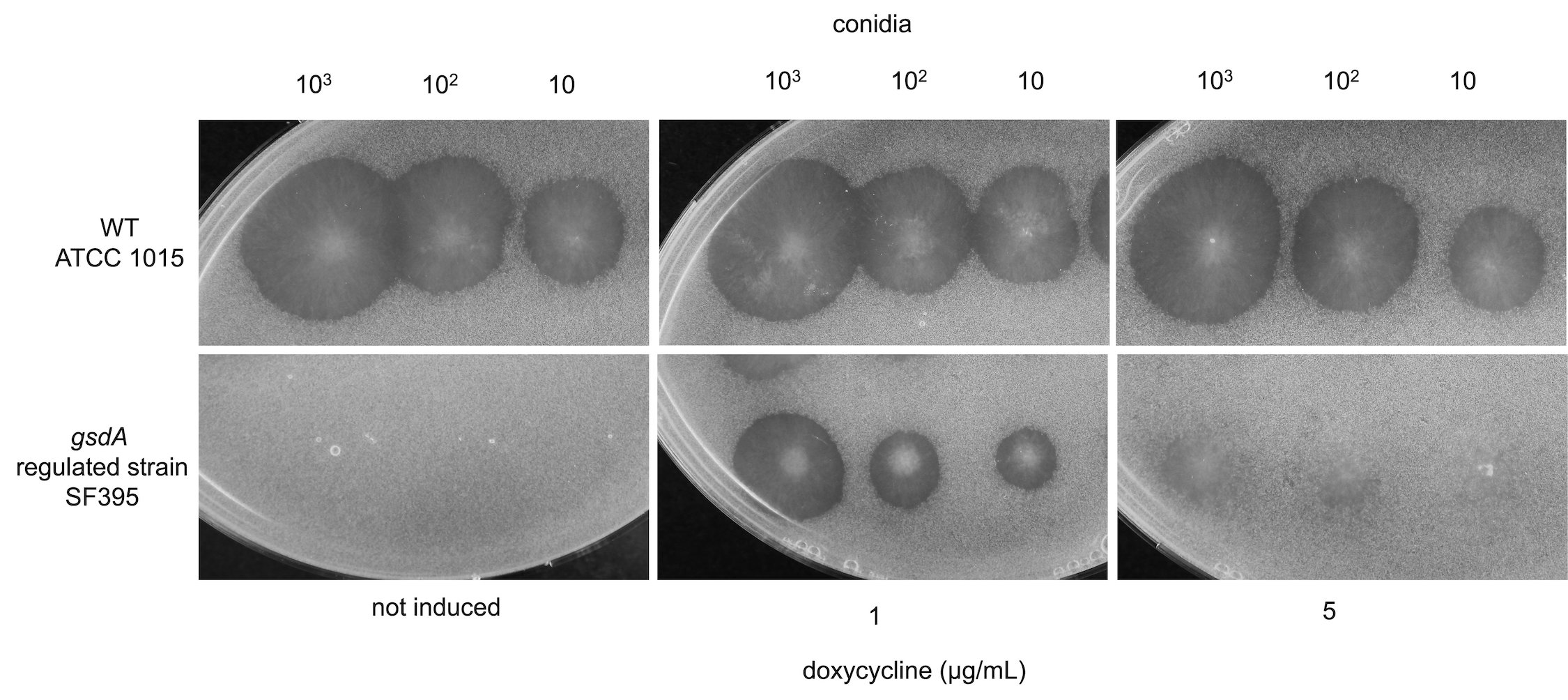

Supplement: S1 Fig — Back side of growth plate presented in Fig 4B showing hyphae spread in the area of the halo. Increasing concentrations of doxycycline were applied to the media to regulate the expression of gsdA under the control of the tet-on promoter system in SF395. Growth plates were incubated at 30°C for 90h. (JPG) [file pone.0321363.s004.jpg]

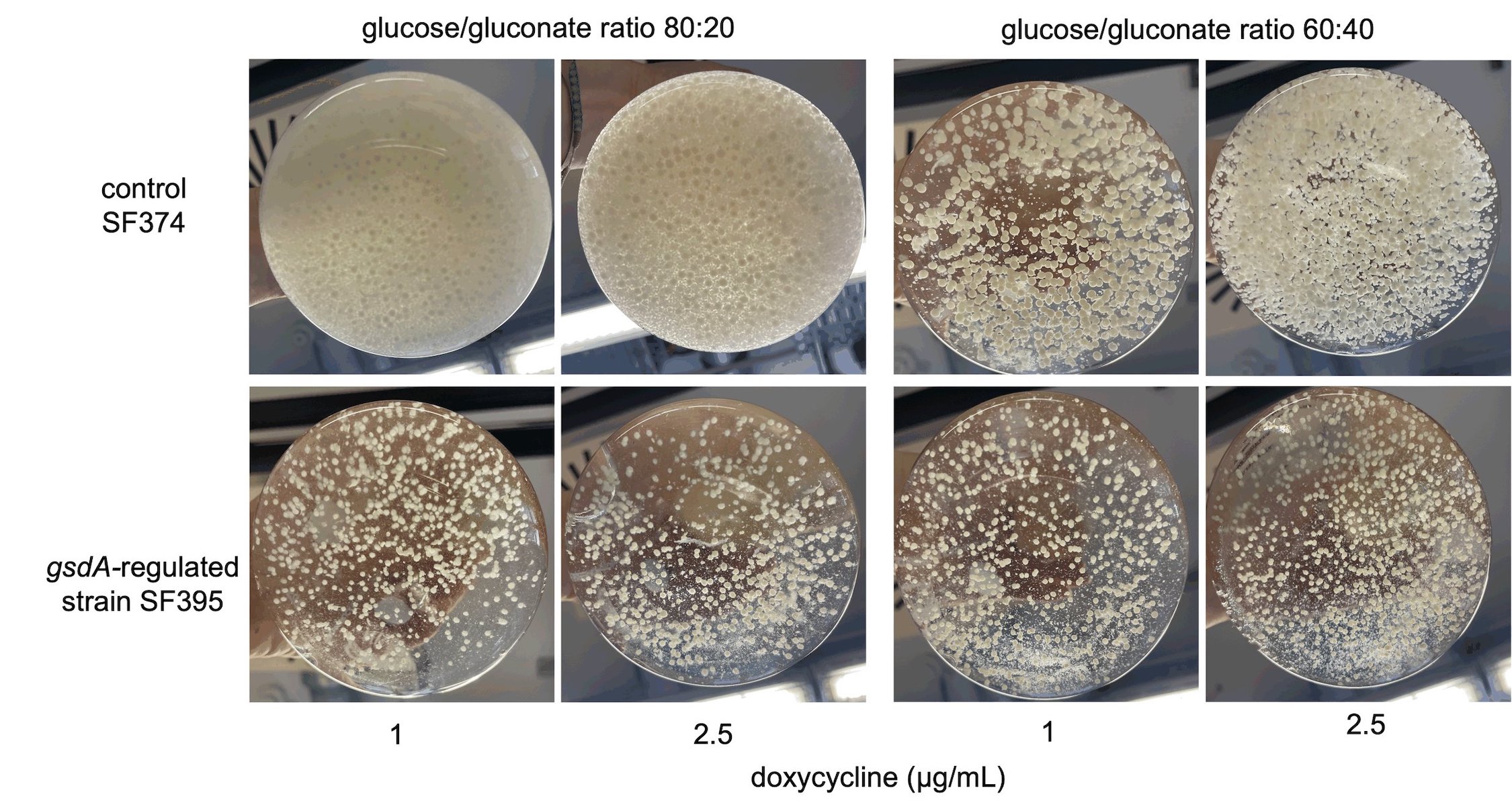

Supplement: S2 Fig — 109 conidia/L of the control strain SF374 or the gsdA-regulated strain SF395 were cultivated in shake flasks with Vogel’s medium with 1 and 2.5 μg/mL doxycycline to induce gsdA under the ptet-on promoter system at the pyrG locus in SF395. Medium contained 20% (w/v) of a glucose/gluconate mixture with a ratio of either (A) 80:20 or (B) 60:40. Images were taken after 120 h of cultivation at 30°C and 200 rpm. (JPG) [file pone.0321363.s005.jpg]

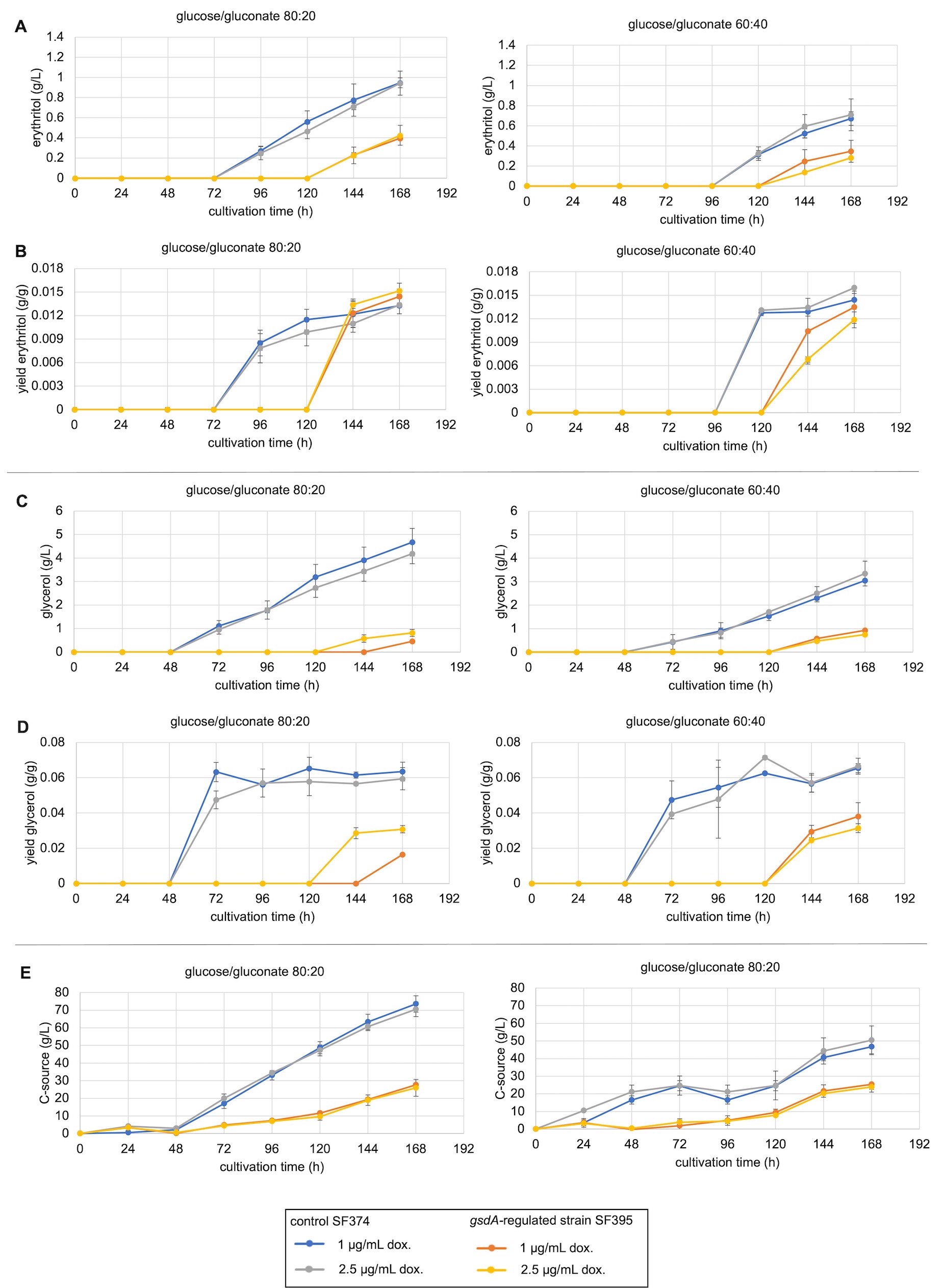

Supplement: S3 Fig — niger with regulated gsdA expression cultivated in medium containing glucose and gluconate. 109 conidia/L of the control strain SF374 or the gsdA-regulated strain SF395 were inoculated in Vogel’s medium with 1 and 2.5 μg/mL doxycycline to induce gsdA under the ptet-on promoter system at the pyrG locus in SF395. Medium contained 20% (w/v) of a glucose/gluconate mixture with a ratio of either 80:20 or 60:40. (A) Erythritol titers and (B) respective yields on consumed carbon source (C-source), (C) glycerol titers and (D) respective yields and (E) C-source consumption were measured in the supernatant of the culture during 168 h of cultivation at 30°C and 200 rpm. (JPG) [file pone.0321363.s006.jpg]
