# Supplementary material for: Regulating the glucose-6-phosphate dehydrogenase encoding gene gsdA and its impact on growth and citric acid production in Aspergillus niger
Source: PLoS One. 2025 Apr 24;20(4):e0321363. doi: 10.1371/journal.pone.0321363 (PMC12021212; doi:10.1371/journal.pone.0321363)

S1 raw image. This gel is the uncropped image that was used to generate Fig 3 of the results. The gel image was generated with a Gel Doc XR+ Gel Documentation System.

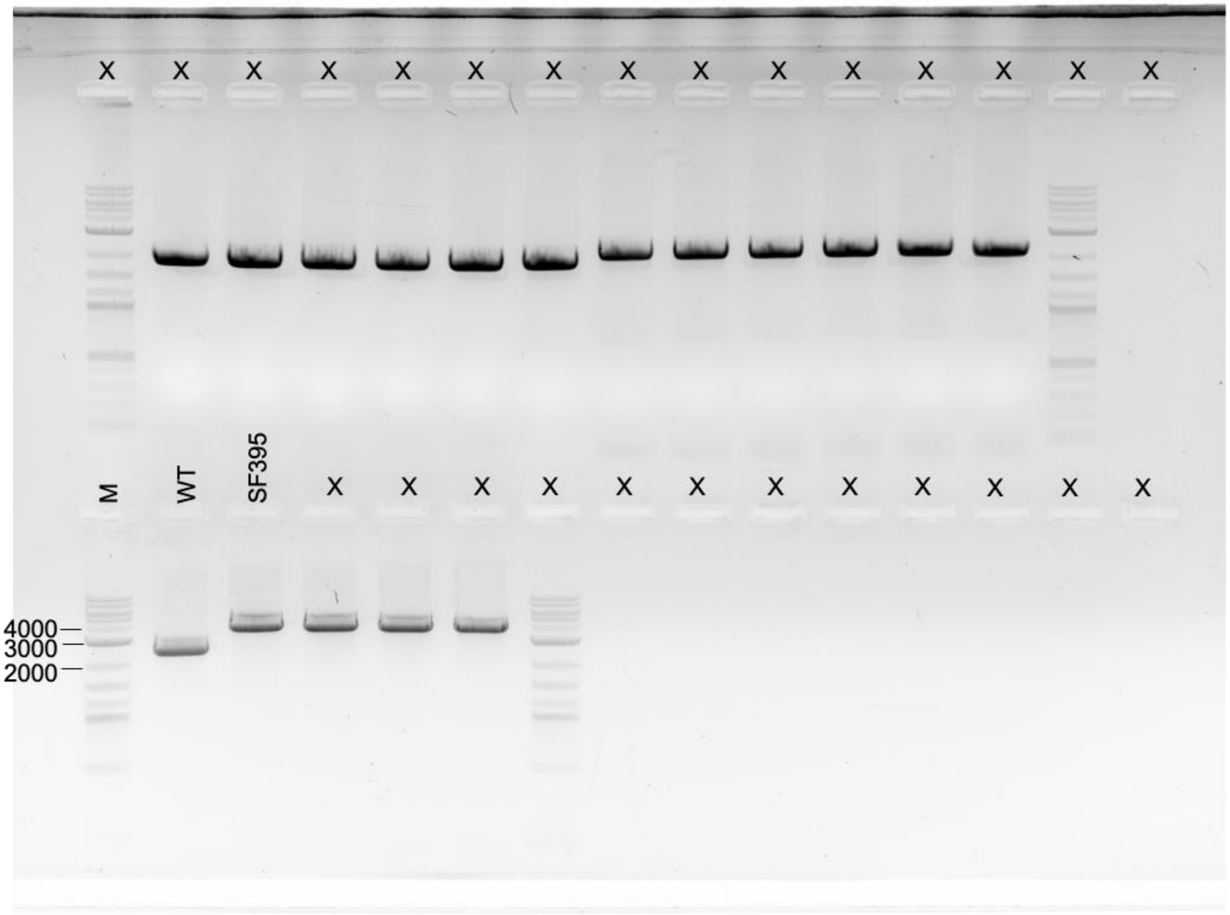

Supplement: S1 raw image — The gel image was generated with a Gel Doc XR+ Gel Documentation System. (PDF) [file pone.0321363.s007.pdf]
