# Supplementary material for: Regulating the glucose-6-phosphate dehydrogenase encoding gene gsdA and its impact on growth and citric acid production in Aspergillus niger
Source: PLoS One. 2025 Apr 24;20(4):e0321363. doi: 10.1371/journal.pone.0321363 (PMC12021212; doi:10.1371/journal.pone.0321363)
Supplement: Attachment — Submitted filename: 250124_Point_by_Point_Fritsche.pdf [file pone.0321363.s009.pdf]

TECHNISCHE UNIVERSITÄT WIEN

Technische Universität Wien
Institute of Chemical, Environmental and Bioscience Engineering
Gumpendorfer Straße 1a, BH01F08
1060 Vienna, Austria

Assoc. Prof. Dr. techn. Matthias Steiger
Head of the Research Group Biochemistry
T +43 1 58801 166552
Matthias.steiger@tuwien.ac.at
www.tuwien.at

Editor-in-Chief
PLOS One

Vienna, January 24 2025

Dear Editor,

We would like to thank both reviewers for the overall positive assessment of the manuscript and the constructive feedback.

As a major change, we measured G6PD in a control strain and an engineered strain, and with this new data we can confirm that our overexpression constructs work as expected. We have also implemented the suggested comments and corrected errors and are now in a position to present an improved version of the manuscript with new data, which we hope will meet with your approval. Changes to the manuscript are in blue color.

Below you will find our detailed response to the reviewers' comments.

With kind regards,

Matthias Steiger
Assoc. Prof. DI Dr. techn.

Head of the Research Group Biochemistry

Technische Universität Wien
Institute of Chemical, Environmental and Bioscience Engineering

Point-by-Point reply to reviewer comments

Reviewer #1: The authors studied to assess the effects of varying the gsdA expression levels in A. niger. While some studies in A. niger had analyzed the impact of varying the gsdA expression levels on NADPH levels and glucoamylase production in A. niger, in this study, the authors studied the effect of overexpressing gsdA on citric acid production in A. niger.
This manuscript includes interesting lines that should be studied, while this is preliminary to be accepted.

Thank you for the valuable feedback and we agree that further work was necessary to further improve the manuscript.

1. First of all, all data need to be statistically analyzed.

We have statistically analyzed our data and included the relevant details of the statistical tests in the data set presented. We have therefore amended Figures 2, 6 and 7 to include information on statistically significant changes.

2. When gsdA is expressed in a tet-on system, the authors need to quantify the gsdA expression levels and NADPH levels in transformants.

We agree that it is necessary to show that our overexpression cassette is functional, and therefore we quantified the specific activity of the induced overexpression strain in comparison to the respective control strain and the non-induced strain. We present the new data in Fig. 2 E and see that the induced strain shows about 3 times higher gsdA activity. This confirms that our expression system is working as expected. The specific activity for the control strain is at the same level as previously reported literature values for this protein (here (Fig. 2 E); Control strain SF374: uninduced: 1,1 U/mg total protein; literature value: 1,0 U/mg total protein, Poulsen et al. 2005,FEBS Fig 3), which shows the consistency of the data presented.

We did not measure NADPH levels as it was shown before (Sui et al. 2020, Micr. Cell. Fact. and Poulsen et al. 2005, FEBS) that although gsdA is overexpressed the NADPH levels show a minor change.

3. **Ln238, Fig. 2B, Please describe that how to messure the glucose concentration.**

Thank you for this comment. Glucose as well as the other secreted metabolites were measured by HPLC analysis. We added further information to the Materials and Methods section to explain in more detail the method used. (L 220 f)

4. **Fig. 2b, c, and d legends do not match the figure.**

Thank you for this valuable comment. The order of compounds was not correct and we corrected the figure legends. In addition, as decribed above Figure 2 was extended with the measurement for specific protein acitivity of G6PD.

5. **Fig. 4b It is hard to understand from the image whether the basal hyphae were spreading or a halo. High-resolution pictures are needed.**
   Can it be said that the lack of halo with adding 5 µg doxycycline is linked to citric acid production? Other organic acid production also needs to be quantified.

Thank you for this valuable comment. We increasd the resolution of the images. Furthermore, we provide (Fig S1) images of the back side of the growth plate for consideration. We can see that the area in which the (also basal) hyphae spread is also the area in which the CaCO3 medium is clear and forms the halo.

Based on your comment we rephrased to sentences to highlight that the halo is only indicative for acidification of the media but does not specifically address citric acid. However, together with the HPLC data presented in Fig 2 we can exclude that a significant amount of other organic acids are formed as no prominent other peak was showing up in the chromatogram at high doxycline concentrations.

With rephrased two sentences in the results section to highlight this circumstance:

L400: The *gsdA*-regulated strain SF395 was spotted on medium composed of glucose and $CaCO_3$ (Fig 4B) and the clearing zone (halo) around the colony was evaluated as an indicator for acidification secreted.

L409: With 5 µg/mL doxycycline the *gsdA*-regulated strain is growing, but no halo is formed under these conditions indicating a reduced acidification of the media, which is in accordance with the previous data (Fig 2) suggesting that increased *gsdA* expression leads to reduced citrate secretion.

6. **Fig. 7b Is the increase in citric acid production versus the concentration of carbon sources between 96-120 h reflecting the reduction of available carbon sources?**
   The authors also need to describe how to quantify carbon sources.

Thank you. Yes we agree it is necessary to show the data for the carbon source consumed, which we included in the S3 Fig panel E. Yes, between 96-120 h of cultivation the carbon source in the medium decreased.

Minor

TECHNISCHE UNIVERSITÄT WIEN

1. **Ln63-65**
   Please describe how much ethanol production has improved in S. cerevisiae.

Thank you! The information is now added to the statement:

In *S. cerevisiae*, engineered NADPH regeneration improved pentose fermentation and production of ethanol from xylitol by 50% [10].

2. **Ln621, DOI may be this, “10.1007/s00253-012-4207-9”.**

Thanks, we corrected the DOI for Citation #35

Reviewer #2: I reviewed the article entitled “Regulating the glucose-6-phosphate dehydrogenase encoding gene gsdA and its impact on growth and citric acid production in Aspergillus niger”.
The authors presented an A. niger strain expressing a ptet-on regulated version of gsdA at the pyrG locus.
The manuscript was beautifully planned and realized. The work is interesting, informative, revealing and deals with an important area of research in the field of PLOS ONE. But I have some suggestions:

Thank you for the valuable feedback and for the overall positive assessment of our work!

1. **In Abstract: The statement “..the split ratio of carbon between glycolysis and the pentose phosphate pathway” is not suitable for metabolism. I think the statement "It determines which metabolic pathway glucose 6-phosphate will go to" would be more appropriate.**

We agree that this is more appropriate and changed the sentence to: “Glucose-6-phosphate dehydrogenase (G6PD) encoded by the gene gsdA is a critical point in the cellular metabolism by determining the metabolic fate of glucose-6-phosphate, directing it either towards glycolysis or the pentose phosphate pathway (PPP).”

2. In Conclusion: It should be written as "G6PD is a crucial metabolic step in central glucose metabolism" instead of "G6PD is a crucial metabolic step in central carbon metabolism".

We agree and changed it accordingly.

3. **In all parts of the article: It should be written as " glucose metabolism" instead of " carbon metabolism". Because other monosaccharides are involved in the metabolic pathway that glucose enters. Again, the carbon source in metabolism is not only monosaccharides. Fatty acids and amino acids also contain carbon.**

TECHNISCHE UNIVERSITÄT WIEN

Thank you. Yes, we changed central carbon metabolism to glucose metabolism in all sentences where it was mentioned

.

4. It is not preferred for primers to have the same bases one after the other. Some primers show the order TTTT, CCCC. How did this affect the results?

Thank you for this comment. Indeed long stretches of repetitive sequences can be problematic. The repetitive sequences mentioned are not directly located on the 3´end of the primer. Only in one case a primer with a triple T on the 3´end was used. The primers worked and yielded the expected PCR products confirmed by fragment sizes, analyzed by gel electrophorese, and sequencing data, Sanger sequencing subcloned fragments.
